# Blunt Cerebrovascular Injury: A Visual Case Report with Computed Tomography Angiography Findings Revealing Carotid and Vertebral Artery Dissection

**DOI:** 10.5070/M5.52229

**Published:** 2026-07-31

**Authors:** Edmund Hsu, Andrew T Kinoshita, Eleanor Chu, C Eric McCoy

**Affiliations:** * University of California, Irvine, Department of Emergency Medicine, Orange, CA; ^ University of California, Irvine, School of Medicine, Irvine, CA; † University of California, Irvine, Department of Radiology, Orange, CA

## Abstract

Blunt cerebrovascular injury (BCVI) is defined as blunt traumatic injury to the carotid or vertebral vessels. Although rare, understanding risk factors and management of possible complications is vital in the emergency department (ED) setting since BCVI is associated with ischemic stroke and potential for poor neurologic outcome or death. In this case, a 27-year-old female patient presented with headache following a motor vehicle collision. Computed tomography angiography (CTA) of the neck revealed a right internal carotid artery dissection. Despite the injury, the patient was largely asymptomatic besides headache and discharged on acetylsalicylic acid (ASA) 81 mg to prevent stroke. Follow-up CTA one week later revealed worsening of the carotid dissection and a new vertebral artery dissection. The patient was admitted and received a diagnostic cerebral angiogram that elucidated the extent of her vascular injuries. The patient was discharged on ASA 325 mg and was followed for 27 months with no further neurological symptoms. Blunt cerebrovascular injury is a potentially life-threatening, yet often underrecognized, injury that occurs in the carotid or vertebral arteries. It is most often associated with high-energy trauma. Up to 80% of patients with BCVI are initially asymptomatic, and effective screening is important for timely diagnosis. Computed tomography angiography is the preferred choice for initial screening and diagnosis of BCVI. Treatment may involve surgical repair, endovascular intervention, and antithrombotic therapy.

**Topics:**

Blunt cerebrovascular injury (BCVI), carotid artery dissection, vertebral artery dissection, computed tomography angiography (CTA), case report.

**Figure:**
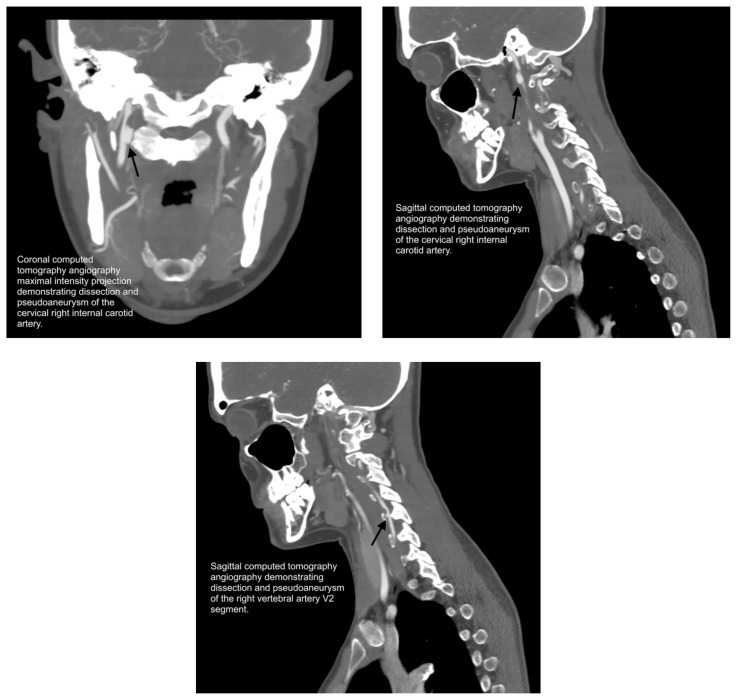
Video Link: https://youtu.be/2nP62QB0zV8

## Brief introduction

Blunt cerebrovascular injury (BCVI) is defined as blunt traumatic injury to the carotid or vertebral vessels.^1^ Blunt cerebrovascular injury is rare, occurring in approximately 1% of patients with blunt head and neck trauma.^2^,^3^ However, understanding warning signs and management of possible complications is vital in the setting of the emergency department (ED), given that BCVI has neurologic morbidity of 37 to 80% and associated mortality of 23 to 40%.^1^,^4^,^5^ Data suggest that ischemic stroke is the main cause of morbidity and mortality from BCVI, which is linked to underdiagnosis or delayed diagnosis.^6^

In this case report, a 27-year-old female patient presented to a level I trauma center with a chief complaint of headache following a motor vehicle collision. Initial computed tomography angiography (CTA) revealed a right internal carotid artery (ICA) dissection. After consultation with trauma and interventional neuroradiology services, the patient was discharged home on low dose acetylsalicylic acid (ASA). Outpatient CTA a week later revealed worsening of the right ICA dissection and a new right vertebral artery dissection. Subsequent admission and diagnostic digital subtraction angiography (DSA) confirmed the two prior lesions, as well as dissections in the left ICA and left vertebral artery. Following summary of the case, we review the risk factors, clinical presentation, preferred imaging modalities in the ED, and treatment of BCVI and its associated complications.

## Presenting concerns and clinical findings

A 27-year-old female patient presented to the ED via ambulance with a chief complaint of headache after a motor vehicle collision. The patient was the front passenger in a 60 miles per hour rear-end collision, which resulted in another passenger in the vehicle being dead upon arrival of emergency medical services (EMS). The patient was placed in a cervical collar by EMS. The patient was amnestic to the events of the accident and unable to recall if she was wearing a seat belt or if airbags deployed. She had repetitive questioning and reported alcohol consumption. She complained of headache and moderate continuous neck pain of an achy quality that was worse with movement.

On exam, the patient’s vital signs were unremarkable. She was in no acute distress, and she was oriented to person, place, time, and purpose with a Glasgow Coma Score of 15. She had a non-focal neurologic exam. Her cervical spine was nontender, and the neck exam did not reveal any wounds, bruises, or bruits. The remainder of her exam was unremarkable. The extended focused assessment with sonography in trauma (eFAST) was negative. After bedside x-rays, the patient underwent computed tomography (CT) for further diagnostic testing.

## Significant findings

### On ED Encounter

Imaging included CT of the head, cervical spine, chest, abdomen, and pelvis, CTA of the neck, and magnetic resonance imaging (MRI) of the brain. Computed tomography angiography of the neck revealed a dissection of the distal cervical right internal carotid artery (ICA) with a 7 mm pseudoaneurysm and 50% luminal narrowing. The remainder of the anterior and posterior neck circulation was within normal limits. There was no vascular occlusion or stroke. The interventional neuroradiology service recommended the patient be discharged with ASA 81 mg daily to prevent ischemic stroke and orders for a 1-week follow-up CTA head and neck.

### On One-Week Follow-Up CTA

Computed tomography angiography of the head was unremarkable. Computed tomography angiography of the neck showed worsening right ICA dissection with increased size of the pseudoaneurysm to 9 mm and worsening stenosis to greater than 70%. Computed tomography angiography of the neck also showed a new dissection of the V2 segment of the right vertebral artery with a 3 mm pseudoaneurysm and greater than 50% stenosis.

## Patient course

At this time, the patient was admitted. She reported episodes of presyncope and had a syncopal episode in the ED. While hospitalized, she received a diagnostic cerebral angiogram to better visualize her vessels and assess the need for intervention. The angiogram revealed the previously seen dissections of the right ICA and right vertebral artery, as well as dissections in the left ICA and left vertebral artery. Given the extent of dissections in multiple vessels, the ASA 81 mg was increased to 325 mg. However, there was no indication for intervention because there was no flow obstruction, and the degree of stenosis was not as severe as it had appeared on CTA. Magnetic resonance imaging during hospitalization showed no evidence of stroke, and the patient had a normal neurological exam. On the morning of discharge, the patient experienced a brief episode of blackening of vision, lasting a few seconds, which was associated with standing immediately upon waking. Symptoms improved with rest. The patient said she was prone to lightheadedness and near fainting, which had been exacerbated since starting spironolactone for acne, and the medication was discontinued along with her desogestrel and ethinyl estradiol combined oral contraceptive pill.

The patient had no new or worsening symptoms at the two-week follow-up. At three-month follow-up, the patient was asymptomatic. Recent magnetic resonance angiography (MRA) of the head and neck with and without contrast showed improvement of findings. Her ASA dosage was decreased to 81 mg and ultimately discontinued at the 9-month follow-up. Her MRA neck imaging at 9 months, 15 months, and 27 months were all negative with resolution of vascular abnormalities. The patient was instructed to return as needed.[Table t1-jetem-11-3-v35]

## Discussion

This case demonstrates the management of an insidious presentation of BCVI. The CTA images and video demonstrate findings discussed in this report. Blunt cerebrovascular injury to the carotid or vertebral vessels is a relatively rare injury that occurs in 0.5 to 3.3% of patients who experience blunt injury, depending on the mechanism of injury.^1^,^2^ In cases of severe head injury, rates of BCVI may be as high as 9%.^7^ Despite relative rarity, BCVI has neurologic morbidity of 37 to 80% and associated mortality of 23 to 40%, making recognition of such injuries important.^1^,^4^,^5^ Vascular wall intimal injuries include, but are not limited to, dissection, intraluminal thrombus, intramural hematoma, pseudoaneurysm, arteriovenous fistula, and transection.^1^

Risk factors for BCVI include altered mental status and Glasgow Coma Scale of ≤8, high-energy mechanism, neck injury including near hanging, cervical spine or basilar skull fracture, and diffuse axonal injury.^1^,^2^,^3^,^8^,^9^,^10^,^11^ Clinical presentation may include focal neurologic deficit or findings unexplained by a diagnosed injury, neck tenderness, cervical bruit, arterial hemorrhage, and expanding cervical hematoma.^1^,^2^,^3^,^12^

Because up to 80% of patients with BCVI may be initially asymptomatic, effective imaging screening is important for timely diagnosis.^6^,^13^,^14^ Computed tomography angiography of the neck is the preferred choice for initial screening and diagnosis of BCVI, showing near equivalence to DSA in diagnostic accuracy.^2^,^6^,^15^ Digital subtraction angiography is the gold standard for diagnosis of BCVI and in this case revealed injury progression a week after the original workup.^16^ However, DSA is not expedient nor widely available because the procedure requires sedation and is invasive.^2^ Risks can include vessel injury and stroke.^2^ Given these limitations, CTA should be utilized initially. Subsequent DSA may be considered when benefits of endovascular intervention are being weighed, as in this case. Other reasons for subsequent DSA may include the need to better characterize a lesion or continued suspicion of BCVI despite a negative CTA.^2^ Magnetic resonance angiography was used for this patient’s long-term follow-up to limit radiation exposure.^2^

Decision to use CTA to screen for BCVI can be based on the modified Denver criteria or additional criteria. Modified Denver criteria is the most commonly used method to assess screening need, yet may miss a significant proportion of cases – up to 30% by some estimates.^13^ A study at a level I trauma center demonstrated a 10% increase in BCVI detection rate with the additional criteria of (1) any suspected cervical spine injury, or (2) any high-energy deceleration trauma impacting the torso (chest, abdomen, or pelvis).^13^

Treatment of BCVI depends on the extent of injury and symptoms and may involve surgical repair, endovascular intervention, and antithrombotic therapy.^7^,^17^ Blunt cerebrovascular injury with vessel transection and signs of active hemorrhage, such as expanding cervical hematoma, require emergent surgery if possible, but often a percutaneous approach is needed.^17^ Neurologic symptoms concerning for stroke should be addressed emergently as well.^17^ For BCVI without active hematoma or acute stroke, prevention of future stroke is the paramount concern. Various meta-analyses have shown no statistically significant difference between anticoagulation therapy and antiplatelet therapy for stroke prevention, and the meta-analyses have deemed the quality of existing evidence as weak.^14^,^18^ In light of this, anticoagulation therapy in the absence of contraindications and in consultation with specialists (e.g. neurology, vascular surgery, interventional neuroradiology, etc.) is often recommended due to significantly lower rates of hemorrhage, greater accessibility, and decreased cost.^14^,^18^

This case study shows the ED evaluation and subsequent follow-up for the management of BCVI. The case highlights the potential progression of injury and complications in the days following the initial insult, despite a more innocuous initial presentation. Key lessons for the emergency medicine provider include the following:

1. In cases of severe blunt trauma, such as motor vehicle collision resulting in another individual’s death, maintain high suspicion of BCVI including in patients with little to no symptoms.
2. Obtain early CTA, which is the diagnostic imaging tool of choice.
3. Consult appropriate services and engage in multidisciplinary management to ensure close follow-up, particularly because early CTA may be negative.
4. Admit or transfer the patient to an institution with appropriate resources if the patient presents with concerns for a BCVI-related complication.

These measures will provide the greatest opportunity to detect BCVI in a timely manner and attenuate the great potential for morbidity and mortality due to this condition.

## Supplementary Information















## Figures and Tables

**Table 1 t1-jetem-11-3-v35:** Timeline of events with notable imaging and recommendations emphasized.

Timepoint	Event
0 days	Motor vehicle collision, initial ED care, no acute distress, headache. CT head, cervical spine, chest, abdomen, and pelvis: No acute findings. **CTA neck:** Dissection of distal cervical right ICA with 7 mm pseudoaneurysm and 50% stenosis. MRI brain: No acute findings. Discharged with **ASA 81 mg** daily and close follow-up.
8 days	**CTA head and neck:** Worsening right ICA dissection with 9mm pseudoaneurysm and >70% stenosis. New dissection of V2 segment of right vertebral artery with 3 mm pseudoaneurysm and >50% stenosis. **Admission to hospital recommended.**
9 – 11 days	Presented again to ED on recommendation for hospitalization. Syncope witnessed in ED. Presyncope during hospitalization. CTA head and neck, MRI brain repeated with no new findings. **DSA:** Previously seen dissections of the right ICA and right vertebral artery. New dissections in the left ICA and left vertebral artery. Discharged with **ASA 325 mg** daily. Spironolactone and combined oral contraceptive discontinued.
24 days	No new or worsening symptoms.
3 months	Asymptomatic. **MRA head and neck:** Improved findings. **ASA 81 mg.**
9 months	**MRA head and neck:** Negative, vascular abnormalities resolved. **ASA discontinued.**
15 months	**MRA head and neck:** Negative.
27 months	**MRA head and neck:** Negative. **Return as needed.**

**Table 2:** Modified Denver criteria plus additional criteria for blunt cerebrovascular injury.

**Modified Denver criteria signs**	Arterial hemorrhage
	Cervical bruit
	Neck hematoma expansion
	Focal neurologic deficit
	Neurologic exam inconsistent with imaging
	Stroke on secondary imaging
**Modified Denver high-risk injury patterns**	**High-energy transfer mechanism with another risk factor below:**
	LeFort II or III fracture
	Cervical spine fracture C1–C3 (superseded below)
	Basilar skull fracture involving carotid canal
	Petrous bone fracture
	Diffuse axonal injury
	Hanging with anoxic brain injury
**Additional risk factor criteria**	Any suspected cervical spine injury (expands C1–C3 criterion)
	Deceleration trauma with torso impact
